# Optimism, Agency, and Success

**DOI:** 10.1007/s10677-018-9894-6

**Published:** 2018-05-13

**Authors:** Lisa Bortolotti

**Affiliations:** 1 Philosophy Department and Institute for Mental Health, University of Birmingham, Edgbaston B15 2TT, UK

**Keywords:** Optimism bias, Dispositional optimism, Agency, Success, Motivation, Positive illusions

## Abstract

Does optimism lead to success? Friends of optimism argue that positive beliefs about ourselves and our future contribute to our fitness and mental health, and are correlated with good functioning, productivity, resilience, and pro-social behaviour. Sceptics, instead, claim that when we are optimistic we fail to react constructively to negative feedback, and put ourselves at risk because we underestimate threats. Thus, it is controversial whether optimistic beliefs are conducive to success, intended as the fulfilment of our goals in a given domain. According to the traditional view, optimistic beliefs lead to success when they do not involve any distortion of reality, and according to the trade-off view, they lead to success when they involve a distortion of reality, but a small one. Based on the literature about positive illusions in the perception of romantic partners and in the assessment of future health prospects, I suggest that optimistic beliefs lead to goal attainment when they support agency by contributing to the sense that we are competent and efficacious agents and that our goals are both desirable and attainable.

## 1 Optimism and Goal Fulfilment

Most of us have optimistic beliefs. For instance, I tend to believe that I am more productive than the average academic and that I will not get cancer. Although the link between optimism and success is widely endorsed in popular culture, it is controversial in the empirical literature, where we find both examples of optimistic beliefs leading to success (e.g., Fox 2013) and examples of optimistic beliefs leading to failure (e.g., Lavallo and Kahneman 2003; Lewine and Sommers 2016).

Let me start with an example of optimistic beliefs linked to success where success is cashed out in terms of *overall social adjustment*. People were found to adapt better to the loss of a spouse when they had unrealistically self-enhancing beliefs, that is, excessively positive beliefs about themselves, their skills, and their talents (Yan and Bonanno 2015). Adjustment was measured by structured interviews and ratings by family and friends where the person’s mental health and quality of social interactions were also taken into account.

Let me turn now to an example of optimistic beliefs that are not conducive to success, where success is measured by *academic performance and engagement*. In the short term, students who had illusory beliefs about their academic ability experienced higher levels of wellbeing than students without self-enhancing beliefs (Robins and Beer 2001). But in the long term, they became progressively less engaged with their academic context, had decreasing self-esteem, and experienced lower levels of wellbeing. In self-enhancers academic performance was no better than in people who had more realistic expectations, and when self-enhancers realised that they could not achieve the grades they expected, they started considering grades less important (the so-called ‘sour grapes’ effect).

As the examples show, the psychological evidence suggests that there is no straight-forward relationship between the optimistic content of our beliefs and goal attainment. However, it is important to identify key features that make some optimistic beliefs but not others conducive to success. Theoretically, the project contributes to a better understanding of the conditions for successful agency. Practically, the project has significant applications for psychological interventions aimed at promoting those forms of optimism that are linked to goal attainment.

In this paper, I challenge the two dominant views about the relationship between optimism and success: (1) optimistic beliefs lead to success when they involve no distortion of reality; (2) optimistic beliefs lead to success when they do involve a distortion of reality, but not a major one. Both views are problematic, because some optimistic beliefs that involve a substantial departure from epistemic rationality and significantly idealise reality lead to success. I will propose a third view, according to which the beliefs leading to success are those that impact on our behaviour, sustaining our motivation to pursue our goals at critical times.

In sections 2 and 3, I describe different ways in which optimism is measured and tested in social psychology, and introduce two broad approaches to the relationship between optimism and success, the *traditional view* and the *trade-off view*. In sections 4 and 5, I consider the effects of optimism in two domains where the role of optimistic beliefs has been studied extensively: perceptions of our romantic partners and attitudes towards our future health prospects. In section 6, I reflect on the cases previously discussed, show that they cannot be easily accounted for by the traditional view or the trade-off view, and propose a third view of the relationship between optimism and success. Independent of how realistic they are, the beliefs linked to success sustain our motivation to pursue our goals notwithstanding the inevitable setbacks and challenges we face.

## 2 Defining and Measuring Optimism

There are two main notions of optimism discussed in the psychological evidence, positive illusions and dispositional optimism. *Positive illusions* are patterns of beliefs about self, world, and future characterised by “systematic small distortions of reality that make things appear better than they are” (Taylor 1989, page 228). There are three basic types of positive illusions. First, we harbour the *illusion of control* when we believe that we can control independent, external events (e.g., Langer and Roth 1975). Second, we are vulnerable to the *illusion of superiority* when we believe that we are better than average in a variety of domains, including attractiveness, intelligence, and even moral character (e.g., Brown 2012; Wolpe et al. 2014). Third, we are prone to the *optimism bias* when we have the tendency to predict that our future will be largely positive and will yield progress, and that negative events will not be part of our lives (Weinstein 1980; Sharot 2011).

Shelley Taylor’s positive illusions are characterised as tendencies to adopt *false beliefs*. For instance, the optimism bias (also called *unrealistic optimism*) is defined as “an *erroneous* belief that personal negative outcomes, assessed on some form of absolute likelihood scale, are less likely to occur than is objectively warranted” (Shepperd et al. 2013, p. 396, my emphasis). An example of optimism bias would be if I underestimate the chances that I will be involved in a car accident during my lifetime. There is also a form of *comparative* optimism bias which occurs “when a person *incorrectly* judges how his or her risk compares with that of other people” or “when people in a sample estimate that they are less likely on average to experience a negative outcome or more likely on average to experience a positive outcome than are their peers” (Shepperd et al. 2013, p. 398, my emphasis). One example would be if I claim that I am much less likely than average to be involved in a car accident, even though I drive just as well and just as often as the average driver.

Although the beliefs we considered are deemed to be *erroneous* or *incorrect*, and Taylor characterises them as (small) *distortions of reality*, it is important to notice that the beliefs do not need to be false. When we say that we are above average in a domain, we are sometimes right about that; and some of the rosy predictions we make about our future will turn out to be accurate. The beliefs we endorse are *illusory* not because they are false, but because they are the result of biased reasoning. For instance, it has been shown that we dismiss new evidence for a negative turn of events, but take on board new evidence for a positive turn of events (Sharot 2011; Sharot and Garrett 2016). As the key epistemic cost is an asymmetrical regard for the relevant evidence rather than inaccuracy as such, the form of optimism exemplified by positive illusions should be labelled as *unwarranted* rather than *unrealistic* (see Jefferson et al. 2017 for a further discussion of this point). That is why I depart from convention and prefer to call the beliefs studied in the positive illusions literature *optimistically biased beliefs* rather than *unrealistically optimistic beliefs*. Moreover, given the nature of the bias that affects our self-related beliefs, it is appropriate to regard them as *epistemically irrational*, in the sense that they may not be well-grounded on existing evidence, and they are generally resistant to new counter-evidence.

Not all forms of optimism are the result of biased reasoning. For instance, *dispositional optimism* is a personality trait exhibited by different individuals to different degrees. It is a generalized tendency to expect positive outcomes (Carver et al. 2010; see also Conversano et al. 2010). Differently from the optimism bias, which is measured by comparing the perceived chance of an event occurring with its objective chance of occurring, dispositional optimism is measured by using the revised Life Orientation Test (Carver et al. 2010; Scheier et al. 1994), which asks people to what extent they agree or disagree with statements such as “In uncertain times, I usually expect the best” or “I rarely count on good things happening to me”. It is not clear what the correlation is between positive illusions and optimistic expectations, as the studies investigating such a relationship have delivered so far inconsistent and inconclusive results (see Shepperd et al. 2013 for a brief discussion of this point).

Dispositional optimism is correlated with active coping or problem-focused coping in a number of different studies (e.g., Scheier et al. 1986). This means that when we are high in dispositional optimism we tend to respond positively and constructively to setbacks, without denying obstacles or being defensive about negative feedback (Conversano et al. 2010; Nes and Segerstrom 2006). As a result, when we are dispositionally optimistic, we have more satisfying and stable relationships and we enjoy better physical and mental health. In the relationship domain, dispositional optimism enables couples to respond more constructively to stressful situations and conflict (Murray and Holmes 1997; Srivastava et al. 2006). In the health domain, dispositional optimism is protective with respect to anxiety and depression, linked to better health outcomes and lower mortality, and conducive to more effective coping in the presence of health threats (Conversano et al. 2010; McKenna et al. 1993). The positive effects of dispositional optimism have been explained by two hypotheses. First, due to being framed broadly, optimistic expectations allow for a reinterpretation of the impact of the available evidence on our chances to attain our goals, enabling us to keep engaged in the face of challenges. Second, due to having a positive content, general expectations are linked to a more balanced emotional profile, reducing stress, supporting behavioural changes, and helping us respond effectively to threats.

There seem to be two main differences between dispositional optimism and positive illusions. First, whereas positive illusions can be ‘switched on and off’ depending on life circumstances and mood, dispositional optimism is a fairly stable personality trait. Second, whereas dispositional optimism has well-documented benefits across the board, positive illusions can have positive or negative effects depending on their applications. Let me consider both features in turn.

Recent studies have shown that we maintain our dispositional optimism in the face of quite dramatic changes in life circumstances, including chronic illness (Fournier et al. 2002). Instead, positive illusions can decrease after adversities, including illness, disability, abuse, and trauma (Bloom 2003). Moreover, there is some evidence that positive illusions can be sensitive to environmental factors: they are enhanced in people who experienced a warm parenting style in their childhood (Peterson 2000; see also Moutsiana et al. 2013). There is also evidence that positive illusions can be controlled, at least to some extent: when we are given more information about the evaluation or the prediction to be made and we are made to feel more accountable for our evaluation or prediction, then we are less vulnerable to causal illusions and self-enhancing biases (e.g., Matute et al. 2015; Sedikides et al. 2002). So, it seems that we are stuck with or without dispositional optimism, but our self-related beliefs can be debiased to some extent. The question is whether it is a good idea to debias them.

Some have argued that positive illusions are the most promising case of *adaptive misbeliefs* (McKay and Dennett 2009, page 507), that is, although they are epistemically problematic, they are also biologically and psychologically adaptive, enhancing our chances of survival and reproduction, and contributing to our wellbeing. Some of the benefits attributed to positive illusions seem to go beyond a mere increase in fitness or wellbeing: positive illusions have been correlated to mastery, motivation, productivity, resilience, and altruistic and caring behaviour (Taylor 1989, page 203; Alicke and Sedikides 2009), suggesting that they are an important factor in supporting mental health and moral conduct. Such considerations may give us a reason to resist debiasing interventions.

However, as we saw, the recent literature has revealed that optimistic beliefs can have harmful as well as beneficial consequences. For instance, optimistic predictions about our future can increase self-confidence and become self-fulfilling in some circumstances, but in other circumstances they lead us to ignore potential obstacles and take unnecessary risks. By believing that the future will be rosy, we become complacent or turn out to be unprepared when failure ensues (Shepperd et al. 2013). This is due to optimism fostering feelings of invulnerability and leading to disappointment when reality does not match our predictions. Such considerations may give us a reason to pursue debiasing strategies.

## 3 The Traditional View and the Trade-Off View

In the empirical literature, there are two broad approaches to the question whether optimism is good for us. What I am going to call the *traditional view* says that having true beliefs contributes to psychological wellbeing, whereas having false beliefs leads to psychological distress. This view is implicit in some accounts of mental distress where ‘madness’ is defined or explained in terms of irrationality (Bortolotti 2013). Moreover, the view underlies some theories about the causes of depression. For Aaron T. Beck, for instance, cognitive errors, including negative automatic thinking, negative self-schemata, and errors in logic, precede symptoms of depression such as low mood (Beck 1967). According to Martin E. Seligman’s learned helplessness theory, we become depressed when, as a result of life experiences, we come to believe that we cannot escape negative events and that there is nothing we can do to change our circumstances for the better (Seligman 1974). In Beck’s and Seligman’s accounts, depression is the result of thinking irrationally and adopting false beliefs.

The trade-off view emerges as an explicit challenge to the traditional view and argues that, in at least some contexts, our psychological wellbeing requires having beliefs that distort reality in a particular way (Taylor and Brown 1988). To be mentally healthy, we need to be optimistic about our skills and talents, our capacity to control external events, and our future. Having true beliefs, instead, can lead to distress. When we are affected by low mood, we tend to have true beliefs about our skills and talents, our capacity to control external events, and our future. This affects us negatively, as it is shown in the depressive realism literature where people with depression are found more realistic in their self-related beliefs than people without depression, due to the latter being vulnerable to “self-enhancing distortions” (Lewinsohn et al. 1980).

What do the two approaches have to say about the link between optimism and success? Although they might not have anything specific to say about success as such, they have predictions to make about the relationship between optimism and psychological wellbeing, which differently from hedonic wellbeing also includes adjustment or good functioning. The traditional view predicts that positive illusions, intended as distortions of reality, are not conducive to good functioning. This prediction cannot explain the robust data about the adaptiveness of positive illusions. The trade-off view predicts that positive illusions are conducive to good functioning because they offer the particular distortion of reality we need to enjoy mental health. But how can we explain then that some forms of optimism are harmful? The trade-off view maintains that optimism ceases to bring benefits when it is *off-the-mark* (Armor and Taylor 1998, 2003) because excessive optimism can prevent us from anticipating set-backs and preparing for negative outcomes (Sweeny et al. 2006; Schacter and Addis 2007). But this does not apply to positive illusions that involve only a *small* distortion of reality.

The problem with both the traditional view and the trade-off view is that, as I previously hinted, it is misleading to make reality distortion the central epistemic fault of positive illusions and the key to their contribution to success. First, some positive illusions give rise to true beliefs but all the beliefs that arise from positive illusions are adopted and retained in a biased (epistemically irrational) way. So the central epistemic fault of positive illusions is their giving rise to beliefs that are unwarranted. Second, for the traditional view only true and rational beliefs are beneficial, and the trade-off view sorts good from bad optimistic beliefs based on whether the distortion of reality they involve is small or large. But some of our optimistic beliefs can be beneficial and conducive to goal attainment even if they significantly idealise reality. We might need a third view.

## 4 Our Love Is Blind

All classic positive illusions are observed in the domain of romantic relationships. We are subject to optimism bias when we underestimate our likelihood of getting a divorce even when we are well-informed about the high divorce rates in the society in which we live (Baker and Emery 1993; Fowers et al. 2001). Optimistically biased predictions about the future of romantic relationships may be supported by other positive illusions, for instance, by the superiority bias and the love-is-blind illusion (Murray et al. 1996a, b). The *relationship superiority bias* occurs when we rate our relationship as better than most (Buunk and van den Eijnden 1997; Rusbult et al. 2000). We experience the *love-is-blind illusion* when we are blind to our romantic partners’ faults, and perceive our partners as better than average in a number of domains including intelligence and attractiveness.

Let me focus here on the common tendency to idealise a partner. Research participants, either dating or married for some time, rated their partners on a list of virtues and faults more positively than the partners rated themselves (Murray et al. 1996a), which was taken to be a significant idealisation because people tend to rate themselves more positively than they ought to. Participants described their partners as having the same qualities they had and as having the same qualities their *ideal* partner would have. In the short to medium term, such idealisations predicted relationship satisfaction better than more realistic evaluations. But what happened in the longer term, when the relationship progressed and partners had more opportunities to ‘show their true colours’?

There are two competing approaches to long-term relationship success. The traditional approach supports a *disappointment* model. Idealising the partner’s qualities does not lead to greater relationship satisfaction in the long run, because when evidence cumulates against the idealised evaluation a more realistic evaluation emerges. The more realistic evaluation is a better basis for devising strategies to cope with the relationship difficulties that are due to the partner’s weaknesses. There is some support for this model: in studies with couples who have been married for a long time the partners who perceived each other more realistically were also the ones who reported to be more satisfied with their relationship (Swann et al. 1994). So, according to the disappointment model, having a realistic view of the partner’s strenghts and weaknesses is better than idealising the partner, because the former frame of mind prepares for the inevitable conflicts that are going to emerge, whilst the latter is destined to cause disappointment.

But there is an alternative model which Sandra Murray and her colleagues endorse, the *self-fulfillment* model, which fits the trade-off view to an extent. This focuses on three effects of partner idealisation. The first is *buffering*: we have a strong sense of security and confidence in the relationship as a result of partner idealisation, and are protected from the potentially disruptive effects of conflict and doubt. The second is *transformation*: we tend to reinterpret our partners’ weaknesses as strengths, and deal with problems rather than distancing ourselves as a result. Here is an example: “Hillary might quell her disappointment in Bill’s stubbornness during conflicts by interpreting it as integrity rather than as egocentrism” (Murray et al. 1996a, page 80). Buffering contributes to sustaining the motivation to pursue relationship satisfaction despite conflicts, because it does not undermine our confidence about the relationship potential; and transformation contributes to our maintaining a positive attitude towards the relationship, because apparent evidence against the partner is reinterpreted in a more optimistic light. Murray and colleagues discuss a third effect of partner idealisation that is more distinctive to this form of optimism, *reflective appraisals*: when partners are idealised, they come to see themselves as we see them, and *live up to our high standards*. This suggests that an optimistic attitude towards our partners can change them for the better: “[I]ntimates can actually turn self-perceived frogs into the princes or princesses they perceive them to be” (Murray et al. 1996b, page 1158).

Further findings by Murray et al. (1996b) are inconsistent with the disappointment model and support the self-fulfilling model. First, in couples with high relationship satisfaction the participants’ evaluations of their partners were “immune to reality” and remained idealised through time, suggesting that no disappointment had ensued as a result of conflict. Second, idealised evaluations of the partner were strongly correlated not only with relationship satisfaction but also with relationship stability. Over time, the idealised evaluations became more realistic, not because people experienced disappointment and lowered their expectations accordingly, but because the partners rose up to the challenge and exhibited the qualities that were initially attributed to them.

The view that Murray and colleagues defend is that, in the long run, due principally to reflective appraisals, the gap between idealisation and reality shrinks. Partners’ evaluations are overly positive to start with but become more accurate as the relationship develops. The conclusion is that “satisfying, stable relationships reflect intimates’ ability to see imperfect partners in idealized ways” and that “intimates who idealized one another appeared more prescient than blind, actually creating the relationships they wished for as romances progressed” (Murray et al. 1996b, page 1155).

In sum, positive illusions can bring success in romantic relationships not because perceptions of the romantic partners are realistic and epistemically rational to start with, but because they have a positive effect on our behaviour in the relationship and support us in the pursuit of our relationship-related goals when problems emerge. Further, perceptions of romantic partners in the studies by Murray and colleagues seem to be significantly idealised and to involve more than a small distortion of reality:

Intimates also appeared to take considerable license in constructing impressions of their partners. Constructed representations—what intimates saw in their partners that their partners did not see in themselves—appeared to reflect their tendency to see their partners as they wished to see them, through the filters provided by their ideals and rosy self-images. (Murray et al. 1996a, page 92).

The idealisation of romantic partners helps us continue to value the relationship as something worth working on, and is linked to more satisfying and more stable relationships in both the short and the long term. This suggests that the perceptions of romantic partners leading to better relationships are those that affect behaviour by increasing resilience.

## 5 Our Future Is Healthy

Next, let me consider how positive illusions impact on lifestyle choices affecting health prospects, and in particular whether they are linked to health-promoting behaviour.

In a now classic study, a counterintuitive result was observed: seropositive men were shown to be significantly more optimistic about not getting AIDS than seronegative men (Taylor et al. 1992). The participants in the experiment showed all three positive illusions: they had an illusion of control over their life events (e.g., “Staying healthy and active will prevent AIDS”), an illusion of superiority manifesting with the attribution of several positive features to themselves (e.g., “My immune system is better than average”), and unwarranted optimism about future health prospects (“I won’t develop AIDS”). Interestingly, such illusions were a response to the threat that seropositivity posed to the participants. Men who had been tested and had decided not to know whether their status was seropositive or seronegative did not show positive illusions to the same extent.

Positive illusions were “associated with reduced fatalistic vulnerability regarding AIDS, with the use of positive attitudes as a coping technique, with the use of personal growth/helping others as a coping technique, with less use of avoidant coping strategies, and *with greater practice of health-promoting behaviors*” (Taylor and Brown 1994, page 24, my emphasis). In the study, people who were more optimistic with respect to their health prospects were also found to be willing to make greater lifestyle changes in order to preserve their health, and the benefits could not be attributed to dispositional optimism because seropositive and seronegative men did not differ with respect to that.

Two common explanations for the link between optimism and health were invoked in this particular case: just like dispositional optimism, unrealistic optimism reduces stress in threat situations, sustaining our motivation to engage in health-promoting behaviour; and it can lead to effective coping strategies, enabling us to face rather than deny or avoid our problems. In the study, *AIDS-specific* unrealistic optimism had more significant effects on *seropositive* than seronegative men. Seropositive men were more likely to look for social support and more likely to believe that they had a control over their future health prospects.

People who have a positive sense of self-worth, belief in their own control, and optimism about the future may be more likely to practice conscientious health habits and to use services appropriately. (Taylor et al. 2000, page 100)

Positive illusions also arise in those of us who have already experienced illness and have to adjust to uncertainty in their health prospects. The sense that we can control our future health prospects has been linked to successful adjustment. For instance, it has been shown that women diagnosed with breast cancer cope better with their difficult situation when they adopt self-enhancing beliefs, such as “I am stronger as a result of the illness” or “I can cope better with cancer than other cancer patients”, and exhibit some illusion of control, such as in the statement: “I am in control of my health condition now” (Taylor 1983; Taylor et al. 1984; Taylor and Sherman 2008). Both in the prevention and the management of illness, positive illusions can contribute to good health outcomes. People seem to behave more responsibly if they believe that it is in their power to avoid or at least delay illness.

In this section, I focused on the contribution of optimistic beliefs to the promotion of attitudes and behaviours related to better health outcomes or better adjustment in people whose health is under threat. Crucially, it is not the realism or epistemic rationality of the adopted belief that predicts its efficacy with respect to health promotion or psychological adjustment. The beliefs reported in the studies were more than just small distortions of reality, as the authors themselves notice discussing the study on women who had breast cancer:

A surprising, somewhat startling finding of the research was that some of the positive beliefs these women developed about their breast cancer experience were illusory (Taylor 1983). Many women expressed the belief that they could personally control the cancer and keep it from coming back. Others insisted they had been cured, although their records showed them to have progressing illness. Despite the fact that these beliefs were inconsistent with objective medical evidence, they were associated with the criteria normally associated with mental health and not with psychological distress. (Taylor et al. 2000, page 99)

The positive outcomes are due to the belief’s role in supporting a conception of the person not as a helpless victim, but as an agent who can determine what her future is going to be. Success in this area means that the agent believes that she can address her health problems, and attempts to solve them by changing her behaviour, without denying existing challenges or threats. Thus, the beliefs that leads to better outcomes are linked to behavioural changes in the face of threats.

## 6 Optimistic People as Better Agents

As we saw in section 3, where we introduced the traditional view and the trade-off view, the former offers an oversimplified account of the relationship between having true beliefs and psychological wellbeing, potentially leading to an unjustified stigmatisation of poor mental health as a manifestation of irrationality. The trade-off view avoids that problem, but in order to explain how optimistic beliefs can be harmful, commits itself to the claim that the optimistic beliefs leading to psychological wellbeing are those that involve just a small distortion of reality.

Both the traditional view and the trade-off view have implications for the goals of psychological interventions. According to the traditional view, psychological health is enhanced when the capacity to have true beliefs is introduced or restored.

The ability to perceive reality as it ‘really’ is is fundamental to effective functioning. It is considered one of the two preconditions to the development of the healthy personality. (Jourard and Landsman 1980, page 75)

According to the trade-off view, instead, psychological health is enhanced when beliefs are distorted *in the right way* and *to the right extent*.

Increasingly, we must view the psychologically healthy person not as someone who sees things as they are but as someone who sees things as he or she would like them to be. Effective functioning in everyday life appears to depend upon interrelated positive illusions, systematic small distortions of reality that make things appear better than they are. (Taylor 1989, page 228)

The studies about romantic relationships and future health prospects reviewed in sections 4 and 5 are not satisfactorily explained either by the traditional view or by the trade-off view. The results we discussed suggest that the beliefs leading to better relationships and better health outcomes are *optimistically biased*, and biased to such an extent that they are likely to *involve a major idealisation of reality* at the time of their adoption. What can explain those results?

Armor and Taylor (1998) propose three criteria for beneficial optimism: we benefit from (a) believing that we have skills or talents that cannot be easily measured or verified; (b) believing that we can control events that can actually be controlled, at least to some extent; and (c) believing in positive outcomes that are not too unrealistic. As a result, the ensuing beliefs are *strategic*, in the sense that optimism is applied selectively; *responsive*, in the sense that optimism is sensitive to the features of the situation; and *bounded*, in the sense that there is a limit to how optimistic they are. By not being easily falsifiable and having a positive, but not implausible, content, strategic, responsive and bounded beliefs are likely to allow transformation without bringing about disappointment, just like the general expectations arising in people who are dispositionally optimistic (Armor and Taylor 1998, page 364). Although this is a thoughtful proposal it is silent about the benefits of major idealisations.

Taylor and Sherman (2008) take a different, more promising, line focusing on the link between optimistically biased beliefs and goal pursuit. It is because we are optimistic about how competent and efficacious we are, and about how desirable and attainable our goals are, that we continue to cherish our goals and pursue them after setbacks.

Believing one has many talents and positive qualities, and more talents and more positive qualities than one’s peers, allows one to feel good about the self and to deal with the stressful circumstances of daily life with the resources conferred by a positive sense of self. As such, these self-enhancing beliefs help people thrive in times of stress that might otherwise leave them dispirited and unable to pursue their goals. (Taylor and Sherman 2008, page 59)

Taylor and Sheeman identify one of the key factors, which is the capacity that some beliefs have to support our sense of agency and motivate us to pursue our goals. If we revisit the studies on romantic partners and health prospects in the light of this reading, we find that optimistically biased beliefs in the relationship domain (such as the idealisation of our romantic partners) are linked to relationship satisfaction and stability when they have a positive effect on our capacity to address challenges. In particular, the belief that the partners share features with us and with our ideal partners sustains our motivation to solve the problems our relationship may be facing. The relationship is viewed as desirable and worth fighting for. Optimistically biased beliefs in the health domain (such as the belief that we are in control of our health, at least to some extent, and are better placed to avoid illness than other people in a similar situation) are linked to effective coping in the presence of threats and to engagement in health-promoting behaviour. Our illusions of superiority and control motivate us to modify our behaviour in order to pursue better health prospects and to regard the goals of better health or illness avoidance as attainable.

When we experience illusions of control and superiority we feel that it is in our power to intervene on what goes on in our lives. We become resilient, addressing the inevitable crises in a constructive way (“I can fix this”). Positive illusions play the role of Albert Bandura’s *self-efficacy* beliefs and as such support goal fulfilment.

People's self-efficacy beliefs determine their level of motivation, as reflected in how much effort they will exert in an endeavor and how long they will persevere in the face of obstacles. The stronger the belief in their capabilities, the greater and more persistent are their efforts (…). Strong perseverance usually pays off in performance accomplishments. (Bandura 1989, page 1175)

Beliefs about self-efficacy and competence are often biased, as we tend to overestimate our capacity to intervene in the environment and to execute the course of action required to attain a given goal (Krueger and Dickson 1994). However, as the psychological literature on self-efficacy suggests, our beliefs underlie various aspects of behaviour that are genuinely *agentic*, such as taking advantage of existing opportunities, creating new opportunities, being assertive in the pursuit of our goals, and being willing to change a situation to better fit our interests, aspirations, and expectations (see for instance, Bandura 2006 and Sadri 1996). Agentic behaviour is correlated with persistence in the pursuit of our goals, and indirectly increases our chances of success.

Positive illusions can translate into effective coping at critical times: we continue to pursue our goals because we believe that we have the capacity to attain our goals, due to self-enhancing beliefs, and illusions of control and superiority. We are willing to devise solutions to our current problems, be these conflicts in the relationship or health threats, because the goals appear to us as still desirable and attainable, due to the optimism bias. Instead of building irrational defences against reality or denying relationship issues and health threats to feel better about our current situation, we acknowledge the problems and take steps to solve them (Boyd-Wilson et al. 2004).

In sum, the link between optimistic beliefs and success cannot be adequately explained in terms of avoiding major distortions of reality. Some optimistic beliefs are realistic or only moderately illusory but fail to play a role in preserving engagement and motivation in the face of challenges. Other beliefs are more than just moderately unrealistic but help sustain changes in behaviour that makes the desired reality come true. If in order to attain our goals we needed our optimistic beliefs to be almost accurate, then it would be difficult to account for the role of partner idealisation in relationship satisfaction, or to explain why seropositive men who believe that their chances of getting AIDS are smaller than warranted by the evidence cope so well with their challenging situation.

In order for us to be successful agents in the face of constant challenges, we need to believe that we can change things for the better, and in order to do that we need to have a sense of competence, control, and efficacy that propels us forward, a sense that our goals are indeed desirable and attainable. The view I am proposing, that optimism leads to success when it supports agency, has implications for psychological interventions. We should encourage the adoption of beliefs about our capacity to control events that are important to us, as such beliefs have a beneficial effect on our perseverance in pursuing the goals that we may initially fail to achieve. Also, we should encourage the adoption of beliefs that emphasise our resources as agents without denying the reality of the setbacks we might experience, as such beliefs are instrumental to enhancing resilience, building our capacity to engage in agentic behaviour at critical times.

## 7 Conclusions

Do optimistic beliefs contribute to success? First, based on recent empirical evidence about the various effects of positive illusions, I argued that not all optimistic beliefs are equally beneficial. Some beliefs about our capacity to control independent events that are important to us help us persevere in pursuing the goals that we initially fail to achieve. Some illusory beliefs that emphasise our resources as agents without denying the reality of the setbacks we might experience are instrumental to our capacity to engage in agentic behaviour at critical times. But other self-enhancing beliefs that boost mood and self-esteem at first, because they give us a sense of invulnerability, may prevent us from adequately preparing for the challenges ahead and lead to failure and disengagement.

As it is the impact of optimistically biased beliefs on behaviour that counts, and not their positive or optimistic content as such, what leads to success is the capacity some beliefs have to sustain our motivation to act in pursuit of our goals. This is indirectly confirmed by the fact that other psychological constructs, distinct from (but related to) optimism, have been developed to explain how our chances of goal attainment are enhanced by sustained motivation. Among these, we find sense of coherence (Antonovsky 1987), hardiness (Maddi 2013), preparedness (Sweeny et al. 2006), and self-affirmation (Hall et al. 2014; Haushofer and Fehr 2014).

The *sense of coherence* consists of three components: “comprehensibility, the extent to which an individual can make sense of adversity; manageability, the extent to which an individual perceives that resources are at her or his disposal to meet the challenges of inordinate demands; and meaningfulness, the extent to which an individual feels that the challenges faced are worth engagement with” (Almedom 2005, page 259). The most obvious overlap with optimism is in the sense that we are sufficiently resourceful and skilled to tackle existing problems (Grevenstein et al. 2016).

Next, *hardiness* is defined as “a pattern of attitudes and strategies that together facilitate turning stressful circumstances from potential disasters into growth opportunities” (Maddi 2013). Hardiness includes a commitment to ourselves and work, a sense of personal control over our experiences and outcomes, and the perception that change represents a challenge and should be treated as an opportunity for growth rather than a threat. Hardy people are curious and engaged, and they see challenges as opportunities to grow and improve (Shepperd and Kashani 1991), being often inspired and motivated to act as a result. The most obvious overlap with optimism can be found in the dimension of control.

A third construct is *preparedness*, that is “a readiness to deal with setbacks and a readiness to take advantage of opportunities” (Sweeny et al. 2006). This notion was inspired by studies suggesting that optimism seems to fade just before we are due to receive feedback or learn about the outcome of our efforts. This is because we need to be ready to respond to uncertainty and brace ourselves for undesired outcomes to avoid disappointment. So, preparedness is sometimes served better by optimism, when we are about to take opportunities and are geared towards pursuing our goals; and sometimes is served better by adopting a more realistic stance, when we need to respond to changes in our environment (Carroll and Shepperd 2009).

Finally, *self-affirmation* is an intervention that has been tested in adverse circumstances that are stigmatising and compromise our decision making. Take poverty: the negative affective states it causes lead us to make short-sighted and risk-averse decisions that are often self-defeating (Haushofer and Fehr 2014). But if we are encouraged to describe past achievements that gave us a sense of pride before making a decision, then our decision making improves and we are more likely to think in ways that show intelligence and flexibility, and to take advantage of the opportunities available to us (Hall et al. 2014). There are interesting parallels between self-affirmation and optimism, among which the emphasis on agency and self-fulfilment.

Although the above constructs are defined and measured in different ways, they are in some cases highly correlated to one another (e.g., Posadzki et al. 2010) and they seem to converge on the positive contribution that self-related optimistic attitudes make to motivation.

In this paper I presented some reasons to believe that optimistically biased beliefs can help us attain our goals by sustaining our motivation to act after we experience setbacks or when some of our cherished goals are under threat. Given that optimistically biased beliefs can be intervened on, should we attempt to eliminate the bias or should we try and instil it in those of us who seem to be immune from it, such as people with depressive symptoms? The current empirical evidence suggests caution. Whereas strategies should be devised to encourage the adoption of beliefs that help us respond contructively to challenges, we should avoid adopting beliefs that make us feel invulnerable. Optimistic beliefs that are unwarranted at the time of their adoption contribute to our chances of success if they support our sense of ourselves as competent and efficacious agents and present our goals as attainable and desirable, because they make us more resilient and persistent in the pursuit of our goals.
